# So-Cheong-Ryoung-Tang Attenuates Pulmonary Inflammation Induced by Cigarette Smoke in Bronchial Epithelial Cells and Experimental Mice

**DOI:** 10.3389/fphar.2018.01064

**Published:** 2018-09-21

**Authors:** Na-Rae Shin, Chul Kim, Chang-Seob Seo, Je-Won Ko, Young-Kwon Cho, Jong-Choon Kim, Joong-Sun Kim, In-Sik Shin

**Affiliations:** ^1^BK21 Plus Project Team, College of Veterinary Medicine, Chonnam National University, Gwangju, South Korea; ^2^Herbal Medicine Research Division, Korea Institute of Oriental Medicine, Daejeon, South Korea; ^3^College of Health Sciences, Cheongju University, Cheongju, South Korea

**Keywords:** So-Cheong-Ryong-Tang, cigarette smoke, iNOS, MMP-9, NF-κB

## Abstract

So-Cheong-Ryoung-Tang is a traditionally used herbal formula for the treatment of pulmonary diseases in China, Korea, and Japan. We investigated the protective effects of So-Cheong-Ryong-Tang water extract (SCWE) in cigarette smoke concentrate (CSC) stimulated human airway epithelial cell line NCI-H292 and mice exposed cigarette smoke (CS) and lipopolysaccharide (LPS). In the CSC-stimulated NCI-H292 cells, SCWE inhibited proinflammatory cytokines in a concentration-dependent manner, as evidenced by a reduction in their mRNA levels. Also, SCWE significant reduced inducible nitric oxide synthase (iNOS) expression and nuclear factor kappa B (NF-κB) phosphorylation in CSC-stimulated cells. The mice were exposed to CS for 1 h per day (a total of eight cigarettes per day) for 7 days and received LPS intranasally on day 5. The mice were administered a dose of SCWE (100 and 200 mg/kg) 1 h before CS exposure. In *in vivo*, SCWE decreased the inflammatory cell count and reduced the expression of the proinflammatory cytokines in the broncho-alveolar lavage fluid (BALF) compared with CS and LPS exposed mice. SCWE attenuated inflammatory cell infiltration in airway induced by CS and LPS exposure, and this decrease was accompanied by a reduction in the expression levels of iNOS and MMP-9 in lung tissue. The extract also inhibited the phosphorylation of inhibitor of kappa B alpha (IκBα) and NF-κB induced by CS and LPS exposure in lung tissue. These results suggest that SCWE may effectively inhibit airway inflammatory responses induced by CS and LPS exposure via the NF-κB pathway. Therefore, SCWE may be a potential treatment for airway inflammatory diseases, such as chronic obstructive pulmonary disease (COPD).

## Introduction

Chronic obstructive pulmonary disease (COPD) is a chronic inflammatory disease of the airway. The pathology of COPD is characterized by neutrophilic airway inflammation, airway remodeling, and pulmonary emphysema in response to cigarette smoke (CS) exposure (Dong et al., 2015; Jung et al., 2016a). CS is a complex mixture of oxidant radicals and various chemical compounds, including reactive aldehydes and semiquinones. CS induces harmful effects on human health and is widely recognized as a primary risk factor for the progression of COPD (Ge et al., 2016). CS exposure leads to airway inflammation, enhanced levels of proinflammatory cytokines and chemokines, and airspace enlargement (Jung et al., 2016b). CS has been shown to increase the infiltration of inflammatory cells such as macrophages and neutrophils that are directly involved in the release of matrix metalloproteinases (MMPs; Lee et al., 2013). MMPs are involved in the development of COPD via modulation of cytokine and chemokine production, extracellular matrix turnover, and tissue remodeling (Cane et al., 2016). MMP-9 has been shown to destroy the normal airway structure and induce airway inflammation. In a clinical trial, MMP-9 was found in the sputum and lavage samples of patients with COPD, and its expression was associated with activation of nuclear factor kappa B (NF-κB), resulting in inflammatory responses (Pang et al., 2015).

Nuclear factor kappa B is a family of transcription factors involved in the regulation of proinflammatory and immune regulatory pathways (Jones et al., 2016). Activated NF-κB induces the expression of many inflammatory mediators, including inducible nitric oxide synthase (iNOS; Kwon et al., 2016). iNOS has been implicated in the pathophysiology of pulmonary disease and is upregulated by various proinflammatory signals. The resulting increase in NO concentration is a part of the immediate immune defense reaction (Hesslinger et al., 2009). Previous studies have demonstrated that iNOS is an important mediator in COPD patients and in the CS-induced mouse model (Hesslingere et al., 2008; Jiang et al., 2015).

So-Cheong-Ryoung-Tang is a traditional oriental medicine and is known as Xiao-Qing-Long-Tang in China and Sho-Seiryu-To in Japan (Park et al., 2015). It is composed a mixture of eight herbal preparations (*Herba Ephedrae, Radix Paeoniae, Fructus Schisandrae, Tuber Pinelliae, Asiasari Radix, Rhizoma Crudus Zingiberis, Ramulus Cinnamomi*, and *Radix et Rhizoma Glycyrrhizae*). We searched electronic databases for relevant studies published before May 2018 in the Oriental Medicine Advanced Searching Integrated System (OASIS). According to the OASIS, this mixture has long been used to treat common cold, bronchitis, and bronchial asthma in the traditional oriental clinic. It is an herbal mixture used to treat various diseases such as allergic rhinitis and asthma (Yang et al., 2009; Wang et al., 2012; Kim et al., 2014; Ku et al., 2016). It was recently shown that So-Cheong-Ryoung-Tang effectively decreased the production of cytokines and chemokines on airway remodeling in allergic asthma model and attenuated allergic effects in a rat model of ovalbumin-induced rhinitis via the phosphatidylinositol 3 kinase (PI3K)/Akt signaling pathway (Kim et al., 2014; Park et al., 2015). However, there is no study available on the effect of So-Cheong-Ryong-Tang on airway inflammation induced by CS and lipopolysaccharide (LPS) exposure.

Therefore, we explored the anti-inflammatory effect of So-Cheong-Ryong-Tang water extract (SCWE) on airway inflammation induced by CS and LPS exposure. To confirm the possible underlying mechanism of SCWE, we investigated the expression and production of inflammatory mediators in a model of CS and LPS induced airway inflammation.

## Materials and Methods

### Plant Materials

So-Cheong-Ryong-Tang water extract consisted of eight herbal medicines, and raw materials were purchased from Omniherb (Yeongcheon, Korea) and HMAX (Jecheon, Korea) in February 2008. The identification of eight raw materials was performed by Prof. Je-Hyun Lee, College of Oriental Medicine, Dongguk University (Gyeongju, Korea). A voucher specimen (2008–KE13–1 ∼ KE13–8) was deposited at the K-herb Research Center, Korea Institute of Oriental Medicine.

### Chemicals and Materials

Coumarin (purity ≥ 99.0%) and cinnamic acid (purity ≥ 98.0%) were purchased from Sigma–Aldrich (St. Louis, MO, United States). Albiflorin (purity ≥ 99.0%), paeoniflorin (purity ≥ 99.0%), cinnamaldehyde (purity ≥ 98.0%), glycyrrhizin (purity ≥ 99.0%), and schizandrin (purity ≥ 99.0%) were purchased from Wako (Osaka, Japan). Liquiritin (purity ≥ 98.0%) was purchased from NPC BioTechnology Inc. (Daejeon, Korea). High-performance liquid chromatography (HPLC)-grade methanol, acetonitrile, and water were purchased from J.T. Baker (Phillipsburg, NJ, United States). Glacial acetic acid (reagent grade) was purchased from Junsei (Tokyo, Japan).

### Preparation of SCWE

So-Cheong-Ryong-Tang water extract decoction was prepared from a mixture of eight medicinal herbs in a ratio as shown in **Supplementary Table S1** (total weight = 3.5 kg, about 93.3 times the composition of a single dose). The mixture was extracted in 35.0 L of distilled water at 100°C for 2 h using an electric extractor (COSMOS-660; Kyungseo Machine Co., Incheon, Korea). The solution was filtered using a standard sieve (No. 270, 53 μm; Chung Gye Sang Gong Sa, Seoul, Korea), evaporated to dryness at 50°C under vacuum using an evaporator Eyela N-11 (Tokyo, Japan), and then freeze-dried using a vacuum control freeze dryer (PVTFD10RS, IlShinBioBase, Yangju, Korea) to give a powder of extract sample. As a result, the amount of lyophilized SCWE powder was obtained 760.6 g (yield: 21.7%).

### HPLC Analysis of SCWE Decoction

The quantitative analysis of the eight biomarker compounds in SCWE decoction was performed using a Shimadzu Prominence LC-20A system (Kyoto, Japan) equipped with LC-20AT pumps, DGU-20A3 online degasser, CTO-20A column oven, SIL-20AC auto sample injector, and SPD-M20A photodiode array (PDA) detector. All chromatographic data were recorded and processed by LCsolutions software (Version 1.24 SP1). The separation of the eight analytes was achieved on a Phenomenx Gemini C18 column (250 mm × 4.6 mm, 5 μm, Torrance, CA, United States), and column temperature was maintained at 40°C. The mobile phase consisted of 1.0% (v/v) aqueous acetic acid (A) and 1.0% (v/v) acetic acid in acetonitrile (B), and gradient conditions were as follows: 15–65% B (0–35 min), 65–100% B (35–45 min), 100% B (45–50 min), and 100–15% B (50–55 min). The flowrate was 1.0 mL/min, and the injection volume was 10 μL. For quantitative analysis, lyophilized 200 mg of SCWE extract was dissolved in 20 mL of distilled water and extracted at room temperature for 20 min using an ultrasonicator (Branson 8510, Danbury, CT, United States) The solution was then filtered through a 0.2 μm membrane filter (PALL Life Sciences, Ann Arbor, MI, United States) before the HPLC injection.

### Cell Culture and Cell Viability

A mucoepidermoid pulmonary carcinoma cell, NCI-H292, was maintained in RPMI 1640 supplemented with 10% heat-inactivated fetal bovine serum (FBS) and antibiotics at 37°C in a 5% CO_2_ incubator and 95% air. The cell viability in response to SCWE was measured using a 3-(4,5-dimethylthiazol-2-yl)-2,5-diphenyltetrazolium bromide (MTT) assay. The NCI-H292 cells were cultured in 96-well plates at a density of 3 × 10^4^ cells/well. The SCWE was added to each individual well at concentrations of 1.25, 2.5, 5, and 10 μg/mL, followed by incubation for 24 h. MTT solution (10 μL) was added to each well, and the cells were incubated for 4 h at 37°C. After incubation, 100 μL of dimethyl sulfoxide (DMSO) was added to each well to solubilize the produced formazan. The optical density was measured at 570 nm using a microplate reader (Molecular Devices Ins, CA, United States).

### Reverse Transcriptase-Polymerase Chain Reaction (RT-PCR)

The cells were seeded on 60 mm dish at a density of 1 × 106cells/well, treated with a non-toxic concentration of SCWE and incubated in the presence of cigarette smoke concentrate (CSC; 20 μg/mL). To investigate the effect of melatonin on TNF-α, IL-6 and IL-1β expression, the cells were treated with SCWE (1.25, 2.5, 5, and 10 μg/mL). The cells were harvested 24 h after SCWE treatment. The total RNA was isolated using TRIZOL^TM^ Reagent (Invitrogen, Carlsbad, CA, United States). For RT-PCR, single-strand cDNA was synthesized from 1 μg of total RNA. The polymerase chain reactions were performed using specific forward and reverse primers (TNF-α, forward, 5′-CAAAGTAGACCTGCCCAGAC-3′, reverse, 5′-GACCTCTCTCTA ATCAGCCC-3′; IL-6, forward, 5′-ATGCAATAACCACCCCTGAC-3′ and reverse, 5′- ATCTGAGGTGCCCATGCTAC-3′; IL-1β, forward, 5′-AGCCAGGACAGTCAGCTCTC-3′ and reverse, 5′-ACTTCTTGCCCCCTTTGAAT-3′; GAPDH, forward, 5′-CAAAAG GGTCATCTCTG-3′, reverse, 5′-CCTGCTTCACCACCTTCTTG-3′). The PCR products were fractionated via 1.5% agarose gels electrophoresis and stained with 5 μg/mL ethidium bromide.

### Animals

Specific pathogen-free male C57BL/6N mice (20–25 g, 6–8 weeks old) were purchased from the Samtako Co. (Osan, Korea). They were housed in groups of nine under standard conditions (temperature 22 ± 2°C, humidity 55 ± 5%, 12-h-light/dark cycle) with food and water available *ad libitum*. All experimental procedures were approved by the Institutional Animal Care and Use Committee of the Chonnam National University.

### Induction of CS and LPS in C57BL/6 Mice and Drug Administration

The CS was generated from a 3R4F research cigarette (Kentucky reference cigarette, University of Kentucky, United States), containing 11.0 mg of total particulate matter, 9.4 mg of tar, and 0.76 mg of nicotine per cigarette. Exposure of CS (one puff/min, 35 mL puff volume over 2 s, every 60 s, eight cigarettes per day) was conducted using a CS generator (Daehan Biolink, Seoul, Korea). The mice received 1 h CS exposure in the exposure chamber (28 cm × 28 cm × 28 cm) for 7 days. LPS (10 μg dissolved in 30 μL distilled water) was intranasally instilled under anesthesia on day 5. SCWE was obtained from the Korea Institute of Oriental Medicine (Daejeon, Korea). SCWE (100 and 200 mg/kg) was administered to mice by oral gavage 1 h before CS exposure for 7 days. The 200 mg/kg of SCWE is corresponding to 12 g/60 kg (60 g/60 kg as dried herbal amount) for human dose. As generally two formulas can be used as clinical daily dose, the dose used in this experiment is reasonable.

### Collection of Broncho-Alveolar Lavage Fluid (BALF)

Forty-eight hours after the last intranasal LPS administration, the mice were sacrificed via an intraperitoneal injection of zoletil 50 (25 mg/kg; Virbac Korea. Co., Seoul, Korea), and a tracheostomy was performed. To obtain broncho-alveolar lavage fluid (BALF), ice-cold phosphate buffered saline (PBS; 0.7 mL) was infused into the lung and withdrawn via the tracheal cannulation. This process was repeated once (total volume 1.4 mL). To determine the differential cell counts, 100 μL of BALF was centrifuged onto slides using Cytospin (Hanil Science Industrial, Seoul, Korea). The slides were dried, and the cells were fixed and stained using Diff-Quik^®^ staining reagent (B4132-1A; IMEB Inc., Deerfield, IL, United States) according to the manufacturer’s instructions. The supernatant obtained from BALF was stored at -70°C for biochemical analysis.

### Measurement of Proinflammatory Mediator in BALF

The proinflammatory mediators in BALF were measured using enzyme-linked immunosorbent assay (ELISA) kits (TNF-α; 558534, IL-6; 555240, IL-1β; 559603, BD Science, San Diego, CA, United States) according to the manufacturer’s protocols. The plates were incubated for 10 min in the dark, and the absorbance was measured at 450 nm in a microplate reader (Bio-Rad Laboratories, Hercules, CA, United States).

### Immunoblotting

The lung tissue was homogenized (1/10 w/v) using a homogenizer in lysis buffer (tissue lysis/extraction reagent that contained a protease inhibitor cocktail). The cells were treated as described in the previous sections and then incubated in the presence of CSC (20 μg/mL) for 24 h. The cells were collected by centrifugation, washed twice with PBS, and suspended using lysis buffer. The protein concentrations of the samples were determined using Bradford reagent (Bio-Rad). Equal amounts of total protein (30 μg) were resolved by 10% sodium dodecyl sulfate (SDS)-polyacrylamide gel electrophoresis (PAGE) and transferred to nitrocellulose membranes. The membranes were incubated with blocking solution (5% skim milk) followed by overnight incubation at 4°C with the appropriate primary antibody. The following primary antibodies and dilutions were used: anti-β-actin (1:2000 dilution; Cell Signaling, Beverly, MA, United States), anti-pIκBα (1:1000 dilution; Santa Cruz Biotechnology, Dallas, TX, United States), anti-I kappa B alpha (IκBα; 1:1000 dilution; Santa Cruz), anti-pNF-κB (1:1000 dilution; Abcam, Cambridge, United Kingdom), anti-NF-κB (1:1000 dilution; Abcam), and anti-iNOS (1:1000 dilution; Santa Cruz). The blots were washed three times with Tris-buffered saline containing Tween 20 (TBST) and then incubated with a 1:10,000 dilution of horseradish peroxidase (HRP)-conjugated secondary antibody (Jackson Immuno Research, West Grove, PA, United States) for 30 min at room temperature. The blots were again washed three times with TBST and then developed using an enhanced chemiluminescence (ECL) kit (Thermo Fisher Scientific, Boston, MA, United States).

### Gelatin Zymography

SDS-PAGE zymography was performed as described in Heussen and Dowdle (1980) to determine gelatinase activities. Briefly, zymogram gels consisting of 10% SDS-PAGE containing 1% gelatin were used as the MMP substrate. The gels were washed in 2.5% Triton X-100 for 1 h to remove SDS and then incubated at 37°C for 16 h in developing buffer (1 M Tris-HCl, pH 7.5 with CaCl_2_). Thereafter, gels were stained with 25% methanol/8% acetic acid containing Coomassie Brilliant Blue. Gelatinase activity was visualized as white bands on a blue background that represented the areas of proteolysis.

### Lung Tissue Histopathology

The lung tissue was fixed in 4% (v/v) paraformaldehyde, embedded in paraffin, sectioned at 4 μm thickness, and stained with hematoxylin and eosin (H&E solution, Sigma–Aldrich) to estimate inflammation.

Immunohistochemical slides were deparaffinized, dehydrated, washed in PBS containing 0.05% tween 20 (PBS-T), and incubated for 20 min at room temperature with goat serum to block non-specific staining. The slides were incubated for 2 h at room temperature with primary mouse anti-mouse MMP-9 antibody (diluted 1:100, Abcam). After incubation, they were washed three times, incubated for 1 h at room temperature with biotinylated secondary antibody, and then incubated with an avidin-biotin-peroxidase complex (Vector Laboratories, Burlingame, CA, United States) for 1 h at room temperature. Then, the slides were washed with PBS-T and incubated with diaminobenzidine (DAB, Abcam) for an additional 5 min.

### Statistical Analysis

The data are expressed as means ± standard deviation (SD). Statistical significance was determined using an analysis of variance (ANOVA) followed by a multiple comparison test with Dunnet’s adjustment. *P*-values < 0.05 were considered significant.

## Results

### HPLC Analysis of SCWE Decoction

**FIGURE 1:**
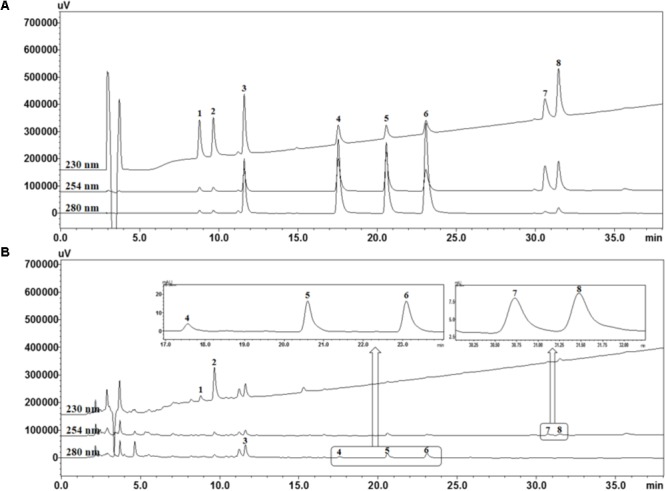
High performance liquid chromatography (HPLC) chromatograms of standard mixtures **(A)** and So-Cheong-Ryoung-Tang water extract (SCWE) decoctions **(B)** at different detection wavelengths. Albiflorin (1), paeoniflorin (2), liquiritin (3), coumarin (4), cinnamic acid (5), cinnamaldehyde (6), glycyrrhizin (7), and schizandrin (8).

For quality assessment of the SCWE decoction, HPLC analysis was conducted using gradient elution of two mobile systems. Nine marker ingredients were completely separated within 35 min; the representative HPLC chromatogram is shown in **Figure 1**. For simultaneous analysis of the SCWE decoction, the detection wavelength of the eight marker compounds was set at 230 nm (albiflorin and paeoniflorin), 254 nm (glycyrrhizin and schizandrin), and 280 nm (liquiritin, coumarin, cinnamic acid, and cinnamaldehyde). The retention times of albiflorin, paeoniflorin, liquiritin, coumarin, cinnamic acid, cinnamaldehyde, glycyrrhizin, and schizandrin were detected at approximately 8.77, 9.65, 11.60, 17.53, 20.57, 23.07, 30.59, and 31.44 min, respectively. Using an established HPLC–PDA method, the amounts of albiflorin, paeoniflorin, liquiritin, coumarin, cinnamic acid, cinnamaldehyde, glycyrrhizin, and schizandrin in the freeze-dried SCWE decoction were found to be 2.19, 11.48, 3.87, 0.19, 0.31, 0.24, 0.69, and 0.52 mg/g, respectively.

### SCWE Reduces Inflammatory Cytokines in CSC-Stimulated NCI-H292 Cells

**FIGURE 2:**
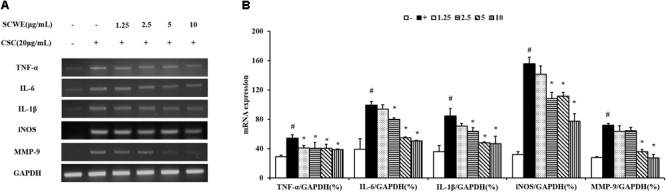
SCWE reduces the expression of inflammatory cytokines in CSC-stimulated H292 cells. **(A)** CSC reduced the mRNA expression of TNF-α, IL-6 and IL-1β. **(B)** Quantitative analysis for mRNA expression. The values are expressed as the means ± SD. ^#^Significantly different from the controls, *P* < 0.05; ^∗^significantly different from the CSC-stimulated cells, *P* < 0.05.

In this study, the ability of non-toxic concentrations of SCWE (1.25, 2.5, 5, and 10 μg/mL) to inhibit the mRNA expression of CSC-stimulated TNF-α, IL-6, and IL-1β. The expression levels of TNF-α, IL-6 and IL-1β increased in CSC-stimulated cells compared to non-stimulated cells (**Figure 2**). The levels of TNF-α, IL-6 and IL-1β decreased in SCWE-treated cells in a concentration-dependent manner compared to the CSC-stimulated cells.

### SCWE Inhibits iNOS and Phosphorylation of NF-κB Expression in CSC-Stimulated NCI-H292 Cells

**FIGURE 3:**
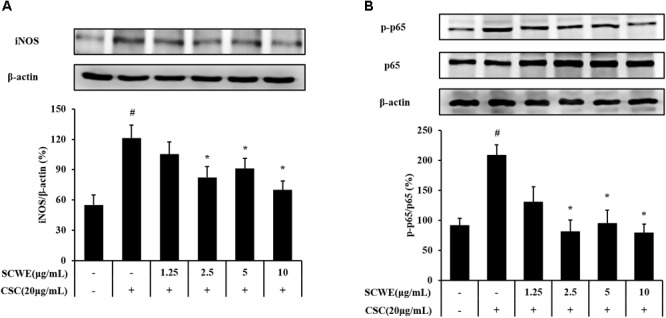
SCWE inhibited the iNOS and phosphorylation of NF-κB expression in CSC-stimulated H292 cells. **(A)** SCWE inhibited the iNOS expression and quantitative analysis for iNOS. **(B)** Phosphorylation of NF-κB expression quantitative analysis for phosphorylation of NF-κB. The values are expressed as the means ± SD. ^#^Significantly different from the controls, *P* < 0.05; ^∗^significantly different from the CSC-stimulated cells, *P* < 0.05.

The iNOS and phosphorylation of NF-κB expression increased CSC-stimulated cells compared to the non-stimulated cells. The iNOS and phosphorylation of NF-κB induced by CSC markedly decreased by SCWE treatment (**Figure 3**).

### SCWE Decreases the Number of Inflammatory Cells in BALF Induced by CS and LPS Exposure

**FIGURE 4:**
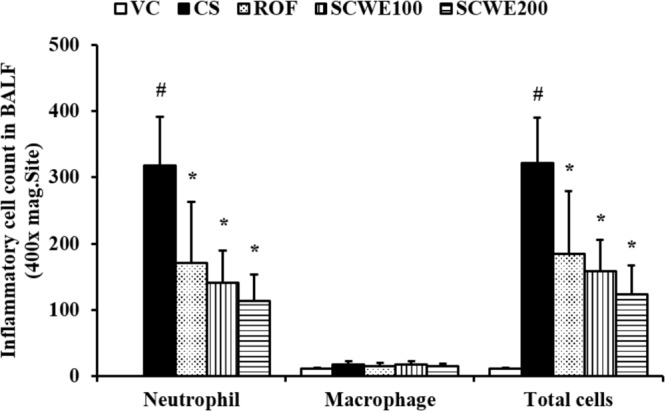
SCWE decreased inflammatory cell accumulation in BALF in CS and LPS exposed animals. VC, vehicle control animals; CS, CS and LPS exposed animals; ROF, roflumilast (10 mg/kg) + CS and LPS exposed animals; SCWE100, SCWE (100 mg/kg) + CS and LPS exposed mice; SCWE200 SCWE (200 mg/kg) + CS and LPS exposed animals. The values are shown as means ± SD. ^#^*P* < 0.05, vs VS; ^∗^*P* < 0.05, vs CS.

The number of inflammatory cells in BALF was increased in CS and LPS exposed mice compared with vehicle control mice. Specifically, CS and LPS exposure markedly increased the number of neutrophils in BALF compared to control. In SCWE treated mice, however, the number of neutrophils in BALF was dose-dependently decreased compared with CS and LPS exposed mice (**Figure 4**).

### SCWE Reduces Proinflammatory Cytokines Induced by CS and LPS Exposure

**FIGURE 5:**
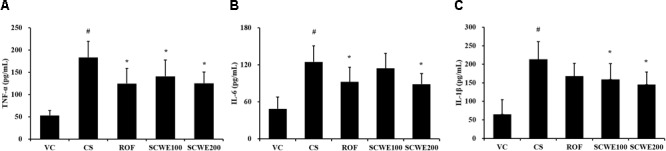
SCWE declined the inflammatory cytokines production caused by CS and LPS exposure. **(A)** Tumor necrosis factor alpha (TNF-α), **(B)** interleukin (IL)-6, and **(C)** IL-1β. VC, vehicle control animals; CS, CS and LPS exposed animals; ROF, roflumilast (10 mg/kg) + CS and LPS exposed animals; SCWE100, SCWE (100 mg/kg) + CS and LPS exposed animals; SCWE200, SCWE (200 mg/kg) + CS and LPS exposed animals. The values are shown as means ± SD. ^#^*P* < 0.05, vs VS; ^∗^*P* < 0.05, vs CS.

In CS and LPS exposed mice, the levels of TNF-α and IL-6 in BALF were significantly increased compared to the vehicle control mice. Roflumilast treated mice significantly reduced TNF-α and IL-6 in CS and LPS exposed mice (**Figure 5**). In addition, SCWE treated mice dose dependently decreased TNF-α and IL-6 levels compared with CS and LPS exposed mice. The results of IL-1β in BALF were similar to those of TNF-α and IL-6. CS and LPS exposure mice significantly increased the level of IL-1β in BALF compared with the vehicle control mice, and SCWE treated mice significantly decreased the level of IL-1β in BALF in CS and LPS exposed mice.

### SCWE Decreases the Expression of Proinflammatory Mediators in Lung Tissue Induced by CS and LPS Exposure

**FIGURE 6:**
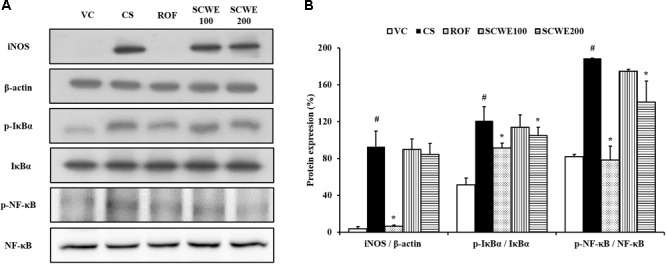
SCWE inhibited inflammatory mediators expression in lung tissue stimulated by CS and LPS exposure. **(A)** Representative figure of protein expression. **(B)** Quantitative analysis for protein expression. VC, vehicle control animals; CS, CS and LPS exposed animals; ROF, roflumilast (10 mg/kg) + CS and LPS exposed animals; SCWE100, SCWE (100 mg/kg) + CS and LPS exposed animals; SCWE200, SCWE (200 mg/kg) + CS and LPS exposed animals. The values are shown as means ± SD. ^#^*P* < 0.05, vs VS; ^∗^*P* < 0.05, vs CS.

Inducible nitric oxide synthase expression was increased in the lung tissue of CS and LPS exposed mice compared to vehicle control mice. Roflumilast treated mice markedly decreased iNOS expression in the lung tissue compared with CS and LPS exposed mice. In addition, SCWE treated mice dose-dependently decreased iNOS expression in lung tissue compared with CS and LPS exposed mice (**Figures 6A,B**).

In comparison to vehicle control mice, the phosphorylation of IκBα was significantly increased in CS and LPS exposed mice. SCWE treated mice markedly and dose-dependently decreased phosphorylation of IκBα in CS and LPS exposed mice. Similarly, NF-κB phosphorylation in lung tissue was markedly increased in CS and LPS exposed mice compared to vehicle control mice. SCWE treated mice significantly decreased in a dose-dependent manner the phosphorylation of NF-κB in CS and LPS exposed mice (**Figures 6A,B**).

### SCWE Attenuates Inflammatory Responses in Lung Tissue Induced by CS and LPS Exposure

**FIGURE 7:**
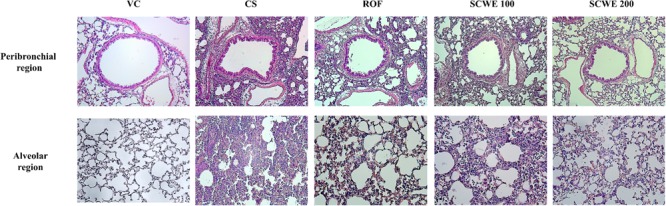
SCWE decreased inflammatory cell infiltration caused by CS and LPS exposure. VC, vehicle control animals; CS, CS and LPS exposed animals; ROF, roflumilast (10 mg/kg) + CS and LPS exposed animals; SCWE100, SCWE (100 mg/kg) + CS and LPS exposed animals; SCWE200, SCWE (200 mg/kg) + CS and LPS exposed animals.

Cigarette smoke and LPS exposed mice exhibited extensive inflammatory cell infiltration into the lung tissue (**Figure 7**). Inflammatory cells mainly accumulating in peribronchial and alveolar lesions. In contrast, roflumilast treated mice decreased inflammatory cell infiltration into lung tissue induced by CS and LPS exposure. Similarly, inflammatory cell infiltration was significantly reduced in a dose-dependent manner in SCWE treated mice compared with CS and LPS exposed mice.

### SCWE Reduces the Expression and Activity of MMP-9 in Lung Tissue Induced by CS and LPS Exposure

**FIGURE 8:**
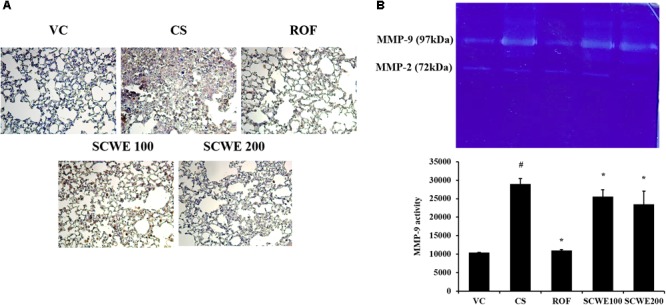
SCWE reduced the expression and activity of MMP-9 stimulated by CS and LPS exposure. **(A)** Representative figure of MMP-9 expression. **(B)** MMP-9 activity. VC, vehicle control animals; CS, CS and LPS exposed animals; ROF, roflumilast (10 mg/kg) + CS and LPS exposed animals; SCWE100, SCWE (100 mg/kg) + CS and LPS exposed animals; SCWE200, SCWE (200 mg/kg) + CS and LPS exposed animals. The values are shown as means ± SD. ^#^*P* < 0.05, vs VS; ^∗^*P* < 0.05, vs CS.

MMP-9 expression in lung tissue was markedly increased in CS and LPS exposed mice compared to vehicle control mice (**Figure 8A**). SCWE treated mice, however, reduced this increased expression of MMP-9 in lung tissue induced by CS and LPS exposure. In zymographs, CS and LPS exposed mice showed a marked increase in MMP-9 activity compared with the vehicle control mice, whereas SCWE treated mice exhibited a marked and dose-dependent reduction in MMP-9 activity compared with CS and LPS exposed mice (**Figure 8B**).

## Discussion

In this study, we evaluated the protective effects of SCWE on airway inflammation induced by CS and LPS exposure. SCWE significantly reduced the elevation of inflammatory cell count and proinflammatory cytokines in BALF induced by CS and LPS exposure. SCWE effectively decreased the enhanced expression of iNOS and phosphorylation of IκBα and NF-κB induced by CS and LPS exposure in lung tissue, which were accompanied by reductions in the inflammatory responses and MMP-9 expression in lung tissue.

Cigarette smoke is a major risk factor for the development and aggravation of COPD characterized by airflow limitation. CS leads to persistent airway inflammation, resulting in irreversible structural changes in lung tissues (Lee et al., 2016). CS was shown to provoke an influx of inflammatory cells, such as neutrophils and macrophages, into the airway (Lee K.H. et al., 2015). Of the inflammatory cells, neutrophils have been associated with the production of various mediators, including cytokines, chemokines, and proteases (Gernez et al., 2010). These responses lead to aggravated airway inflammation and destroyed normal alveolar structure. In this study, SCWE significantly reduced inflammatory cell counts in a dose-dependent manner in CS and LPS exposed mice, and this reduction was accompanied by a reduction in the levels of TNF-α, IL-6, and IL-1β in BALF and CSC-stimulated H292 cells. In addition, SCWE effectively attenuated airway inflammation induced by CS and LPS exposure. Taken together, these results suggest that SCWE may suppress airway inflammation induced by CS and LPS exposure.

The protease MMP-9 has an important role in various physiological and pathological processes (Hernández-Montoya et al., 2015). In development of COPD, MMP-9 was shown to be activated by various stimuli, such as CS, oxidative stress, and other airway proteases in the airway, and was involved in the destruction of normal alveolar structure, production of proinflammatory mediators, and inflammatory cell migration (Cane et al., 2016; Grzela et al., 2016). In a clinical trial, patients with COPD had increased expression of MMP-9 in their sputum and lavage (Mercer et al., 2005). MMP-9 expression is closely associated with activation of NF-κB, which is involved in the process of airway inflammation (Pang et al., 2015). NF-κB is also involved in the regulation of proinflammatory and immune regulatory pathways (Jones et al., 2016). Normally, NF-κB is bound to IκBα in the cytosol in an inactive form, and upon degradation of IκBα by selective ubiquitination, NF-κB is activated (Kwon et al., 2016). Activated NF-κB finally produces MMP-9 and inflammatory cytokines (Zhang et al., 2016). Recently, a study showed that the expression of MMP-9 along with NF-κB was increased in CS extract stimulated peripheral blood mononuclear cells (PBMCs) and murine alveolar macrophage cells and in a CS-induced emphysema rat model (Hou et al., 2015; Li et al., 2015). In addition, NF-κB activation was demonstrated to be required for the increase in iNOS expression involved in the pathogenesis of lung injury (Li et al., 2014). iNOS expression is a key molecule that protects lungs from inflammation (Roh et al., 2010). iNOS expression is upregulated by various proinflammatory signals, resulting in elevations in NO concentrations (Hesslinger et al., 2009). NO production, an active player in the respiratory system and disease, is considered a potential mediator of airway responsiveness (Jiang et al., 2006). In this study, CS and LPS exposed mice exhibited marked increases in MMP-9 and iNOS expression in lung tissue, which were accompanied by elevations in the phosphorylation of NF-κB and IκBα. Importantly, SCWE treated mice significantly decreased the expression of MMP-9 and iNOS with reduction the phosphorylation of NF-κB and IκBα in lung tissue in CS and LPS exposed mice. Also, SCWE reduced expression of iNOS and phosphorylation of NF-κB in CSC-stimulated cells. These results indicate that the protective effect of SCWE is closely associated with the suppression of the NF-κB pathway, which resulted in the reduction in expression of MMP-9 and iNOS.

The traditional herbal formula So-Cheong-Ryoung-Tang is a mixture of eight herbal preparations (**Supplementary Table S1**) and is used to treat pulmonary diseases such as bronchitis, asthma, and cough (Ko et al., 2004; Lee M.Y. et al., 2015). Pharmacological studies have demonstrated that So-Cheong-Ryoung-Tang possesses protective effects against airway inflammation, nasal allergy, pulmonary fibrosis, and asthma (Kao et al., 2000; Das et al., 2009; Yang et al., 2010). In a recent study, So-Cheong-Ryoung-Tang effectively suppressed the production of MMP-9 in human bronchial epithelium cell line BEAS-2B stimulated with TNF-α and IL-4. In a murine model of ovalbumin-induced allergic asthma, SCWE reduced the levels of IL-17 and granulocyte/macrophage colony-stimulating factor (GM-CSF) with reduction in the level of IL-4, which eventually decreased inflammatory cell infiltration and airway remodeling resulting clinical signs of allergic asthma (Kim et al., 2014). This anti-inflammatory properties evidenced by previous studies were strongly consistent with our results. In addition, anti-inflammatory properties of SCWE are associated with not only IκBα/NF-κB signaling but other signaling such as MAPKs. In particular, MAPKs play an important player in the inflammatory responses such as the production of proinflammatory cytokines and chemokines (Turner et al., 2014). However, SCWE has been reported to increase the MAPKs expression in the literature to date (Hwang et al., 2013; Yim et al., 2015). SCWE induces apoptosis via enhancement of MAPK signaling pathway in cancer cells and affects GI motility by the modulation of pacemaker activity through MAPKs in interstitial cells of Cajal. But, previous studies are performed under specific condition including cancer cells without stimulation materials such as LPS, CSC, etc., and *ex vivo* experiments. In addition, anticancer effect of SCWE was induced by the alteration of its active components by bacterial fermentation (Yim et al., 2015). There is no precise description of what reaction will occur under disease conditions such as asthma and COPD until now. Therefore, further research clearly needed to determine the effects of SCWE on MAPKs expression under airway inflammation.

## Conclusion

The present study showed that SCWE suppresses the production of inflammatory mediators and MMP-9 activity in CS and LPS induced mice and CSC-stimulated H292 cells. These effects may be associated with the inhibition of CS-mediated IκBα/NF-κB signaling. Therefore, SCWE is a potential treatment for lung disease, such as CS induced lung inflammation.

## Ethics Statement

All procedures were performed in compliance with the Guide for the Care and Use of Laboratory Animals of the National Institutes of Health and Korean National Laws for Animal Welfare.

## Author Contributions

N-RS and I-SS conceived and designed the experiments. N-RS and J-WK performed the experiments. J-SK, CK, and Y-KC analyzed the data. J-SK, J-CK, and C-SS contributed reagents, materials, and analysis tool. N-RS wrote the manuscript.

## Conflict of Interest Statement

The authors declare that the research was conducted in the absence of any commercial or financial relationships that could be construed as a potential conflict of interest.
