# Supplementary material for: So-Cheong-Ryoung-Tang Attenuates Pulmonary Inflammation Induced by Cigarette Smoke in Bronchial Epithelial Cells and Experimental Mice
Source: Front Pharmacol. 2018 Sep 21;9:1064. doi: 10.3389/fphar.2018.01064 (PMC6160558; doi:10.3389/fphar.2018.01064)
Supplement: Supplementary file 1 [file Table_1.DOCX]

Table 1. Composition of So-Cheong-Ryoung-Tang water extract formula.

| Herbal medicine | Scientific name | Supplier | Family | Origin | Amount (g) | Ratio (%) |
| --- | --- | --- | --- | --- | --- | --- |
| Ephedrae Herba | *Ephedra sinica* Stapf | HMAX | Ephedraceae | China | 5.625 | 15.0 |
| Paeoniae Radix | *Paeonia lactiflora* Pallas | Omniherb | Paeoniaceae | Hwasun, Korea | 5.625 | 15.0 |
| Schisandrae Fructus | *Schisandra chinensis* Baillon | Omniherb | Schisandraceae | Mungyeong, Korea | 5.625 | 15.0 |
| Pinelliae Tuber | *Pinellia ternate* Breitenbach | HMAX | Araceae | China | 5.625 | 15.0 |
| Asiasari Radix | *Asiasarum sieboldi* Miquel var. seoulense Nakai | HMAX | Aristolochiaceae | China | 3.750 | 10.0 |
| Zingiberis Rhizoma Crudus | *Zingiber officinale* Roscoe | Omniherb | Zingiberaceae | Taean, Korea | 3.750 | 10.0 |
| Cinnamomi Ramulus | *Cinnamomum cassia* Presl | HMAX | Lauraceae | Vietnam | 3.750 | 10.0 |
| Glycyrrhizae Radix et Rhizoma | *Glycyrrhiza uralensis* Fischer | HMAX | Leguminosae | China | 3.750 | 10.0 |
| Total |  |  |  |  | 37.500 | 100.0 |
